# Local Variability in Microbiome Composition and Growth Suggests Habitat Preferences for Two Reef-Building Cold-Water Coral Species

**DOI:** 10.3389/fmicb.2020.00275

**Published:** 2020-02-21

**Authors:** Leila Chapron, Franck Lartaud, Nadine Le Bris, Erwan Peru, Pierre E. Galand

**Affiliations:** Sorbonne Université, CNRS, Laboratoire d’Ecogéochimie des Environnements Benthiques (LECOB), Banyuls-sur-Mer, France

**Keywords:** *Lophelia pertusa*, *Madrepora oculata*, DNA, bacteria, skeletal growth rates, Mediterranean canyon

## Abstract

Cold-water coral (CWC) ecosystems provide niches and nurseries for many deep-sea species. *Lophelia pertusa* and *Madrepora oculata*, two cosmopolitan species forming three dimensional structures, are found in cold waters under specific hydrological regimes that provide food and reoxygenation. There is now more information about their feeding, their growth and their associated microbiome, however, little is known about the influence of their habitat on their physiology, or on the composition of their bacterial community. The goal of this study was to test if the habitat of *L. pertusa* and *M. oculata* influenced the hosts associated bacterial communities, the corals’ survival and their skeletal growth along the slope of a submarine canyon. A transplant experiment was used, based on sampling and cross-redeployment of coral fragments at two contrasted sites, one deeper and one shallower. Our results show that *M. oculata* had significantly higher skeletal growth rates in the shallower site and that it had a specific microbiome that did not change between sites. Inversely, *L. pertusa* had the same growth rates at both sites, but its bacterial community compositions differed between locations. Additionally, transplanted *L. pertusa* acquired the microbial signature of the local corals. Thus, our results suggest that *M. oculata* prefer the shallower habitat.

## Introduction

Scleractinian cold-water corals (CWC) such as *Lophelia pertusa* and *Madrepora oculata*, iconic engineer species of submarine canyons, are important frame-builders that provide shelter for a large diversity of associated fauna (Buhl-Mortensen et al., 2010). During the last decades, significant efforts have been dedicated to characterize CWC’s feeding, reproduction, growth, and their associated coral microbiome (Waller and Tyler, 2005; Tsounis et al., 2010; Lartaud et al., 2014; Meistertzheim et al., 2016; Galand et al., 2020). However, due to the difficulty of sampling in the deep sea, a detailed knowledge of their ecology is still lacking when compared to their shallow water counterparts. Such knowledge is important as these animals with a great ecological value are threatened by trawling, pollution and climate change (Freiwald et al., 2004; Foley and Armstrong, 2010).

*Lophelia pertusa* (synonymized recently as *Desmophyllum pertusum* (Addamo et al., 2016) and *M. oculata* are the most abundant species forming reef frameworks in the North Atlantic, the Gulf of Mexico, and the Mediterranean Sea (Freiwald et al., 2004, 2009; Roberts et al., 2006). While *L. pertusa* dominates in many regions, *M. oculata* is often found as a secondary species together with *L. pertusa*. *M. oculata* have fragile branches and it is therefore thought that their framework building capacity is limited (Freiwald, 2002). The relative abundance and/or spatial distribution of *L. pertusa* and *M. oculata* change following geographical locations, which suggests different living strategies (Freiwald et al., 2004; Schröder-Ritzrau et al., 2005; Lartaud et al., 2019). The two species exhibit different responses to environmental changes (Naumann et al., 2014), different skeletal growth rates (Lartaud et al., 2014), reproductive cycles (Waller and Tyler, 2005), feeding strategies (Tsounis et al., 2010; Gori et al., 2014; Galand et al., 2020), and bacterial community associations (Hansson et al., 2009; Meistertzheim et al., 2016; Galand et al., 2018, 2020), with a more stable microbiome for *M. oculata*.

Corals live in very close association with communities of microbes forming together a holobiont. Studies of shallow coral species suggest that the coral microbiome, dominated by photosynthetic zooxanthellate algae, is involved in a variety of physiological functions (Bourne et al., 2016). For CWCs living in a cold and deep environment, without symbiotic zooxanthellae, the role of the associated bacterial community may be different. Recent efforts have been dedicated to characterize the bacterial communities associated to CWCs, to identify their potential role in the nutrition and health of the holobiont, and to assess their responses to environmental changes (Neulinger et al., 2008; Jensen et al., 2019; Kellogg, 2019). Specific bacteria have already been identified such as *Spirochete*s or *Endozoicomonas*, which are observed in *L. pertusa* or *M. oculata* (Kellogg et al., 2009; Meistertzheim et al., 2016), and which may be crucial to the host physiology (Neave et al., 2016). It has also been demonstrated that *L. pertusa* and *M. oculata* have different species-specific microbial communities that can be maintained across different locations (Schöttner et al., 2012; Kellogg et al., 2017; Jensen et al., 2019). In addition, the bacterial community composition of *L. pertusa* can exhibit a strong temporal variability, but also differences within colonies, and between polyps from the same colony, contrary to the *M. oculata* microbiome, which appears much more stable (Meistertzheim et al., 2016; Galand et al., 2018). However, it is still unclear how those corals acquired their microbiome (e.g., vertical or horizontal transfer) and what is the role of the local environmental conditions in the bacterial community dynamic. Studies in aquaria are difficult to conduct to treat this subject as CWCs in captivity change their bacterial community composition with different dynamics seen between species (Galand et al., 2018); analyses *in situ* remain thus fundamental, but transplant experiments have never been conducted for cold water coral microbiomes.

The general aim of this study was to test how the local habitat of *L. pertusa* and *M. oculata* impact their bacterial associations, together with other integrative parameters such as coral calcification and mortality. The study was based on *in situ* transplant experiments with sampling and redeployment of coral fragments at two contrasted sites, one deeper and one shallower, in the Lacaze-Duthiers canyon (LDC) where *L. pertusa* and *M. oculata* reefs are naturally abundant (Fiala et al., 2010; Gori et al., 2013; Lartaud et al., 2014). This multidisciplinary approach ultimately aimed at better understanding the adequacy between CWC and their surrounding environmental conditions, and thus to better assess their resilience to a changing ocean.

## Materials and Methods

### Study Sites

The Lacaze-Duthiers canyon, part of the Gulf of Lion Nature Marine Park, is located in the Gulf of Lion, in the northwestern part of the Mediterranean Sea. The present study focused on two different morpho-bathymetric sites of the canyon: the deeper “A” site (42°32.43N, 03°25.17E), at 530 m depth, is characterized by large colonies of *L. pertusa* and some smaller *M. oculata* colonies that grow on hard substrates outcropping the floor (Lartaud et al., 2017). The shallower “B” site (42°33.47N, 03°24.29E), is located closer to the head of the canyon, where numerous *M. oculata* colonies, together with *L. pertusa*, grow on vertical cliffs that extend from 300 to 480 m depth. This submarine canyon, is characterized by the presence of episodic dense water shelf cascade events driven by wind conditions (Canals et al., 2009). Three different types of events are described, corresponding to autumnal storm events (stratified water masses with low intensity), winter storm events (large amount of particles), and winter cascading events (*non*-stratified water masses with high intensity) (Canals et al., 2006). These events lead to ventilation, changes in the temperature, current speed and transport of material such as organic matter and sediments. These events do not have the same strength at the deeper site compared to the shallow site.

### Coral Sampling and Transplant Procedure

*Lophelia pertusa* and *M. oculata* were sampled from the deeper sites “A” at 530 m and the shallower site “B” at 380 m using a remotely operated vehicle (ROV) deployed form the R/V Minibex Vessel (COMEX SA). Coral fragments were collected in thermally insulated polypropylene boxes to maintain ambient seawater temperature (13°C) during transport to the surface. On board, colony fragments were transferred into aerated 30 L seawater tanks maintained at 13°C using a chiller. The same day, once in the laboratory, the apical parts of corals from both sites were cut with sharp pliers into different nubbins (nubbins with ∼5 polyps for *L. pertusa* and ∼10 polyps for *M. oculata*) and glued on cobblestones with an aquatic epoxy resin to form different transplants units (see Lartaud et al., 2014). Gloves were always used to avoid contamination and pliers were rinsed with alcohol and distilled water between each nubbin handling. Nubbins were kept in aquarium over the night until redeployment the following day. Two *in situ* experiments were performed but all samples were treated the same way. The first focused on coral colonies from both species collected at site B and redeployed on site B (control transplant unit called BB) or on site A (called BA) for 9 months. The second experiment was conducted with corals collected on site A and redeployed on site A (control called AA) or on site B (called AB) for 3 months. A total of twenty-three transplant units were used for *L. pertusa* (BB = 8, BA = 4, AA = 6, and AB = 5) and forty-three for *M. oculata* (BB = 10, BA = 4, AA = 18, and AB = 11). Corals from the transplant unit BA were not collected for the coral microbiome characterization due to the low number of polyps available. When transplant units were retrieved, all nubbins were flash frozen in liquid nitrogen after macroscopic analyses (for mortality and growth rate measures).

### Mortality and Growth Rates

Each coral fragment was photographed on the cobblestones before and after deployment with the same orientation for macroscopic comparisons. Living, dead and newly formed polyps were identified and measured with a caliper following the protocols described in Lartaud et al. (2017) to determine the mortality, budding rates and linear extensions.

### DNA Extractions and Sequencing

For *Lophelia pertusa*, DNA was extracted from the polyps of eight different colonies, and for *M. oculata*, from seven different colonies. In total, the study is based on 23 DNA samples for *L. pertusa* and 25 for *M. oculata*. For the DNA extraction, individual polyps were crushed using a hammer and the tissues were homogenized in tubes containing a garnet matrix without the large ball (Lysing Matrix A) and lysed mechanically using a FastPrep Instrument (MP, Biomedical, Santa Ana, CA, United States). DNA was extracted using the Maxwell Blood DNA Purification Kit LEV and the Maxwell 16 MDx Instrument (Promega, Madison, WI, United States). DNA concentrations were measured by spectrophotometry directly after extractions (NanoDrop ND-1000, Thermo Fisher Scientific, Waltham, MA, United States).

The V1–V3 region of the bacterial 16S rRNA genes were amplified using the primers 27F AGRGTTTGATCMTGGCTCAG modified from Lane (1991) and 519R GTNTTACNGCGGCKGCTG modified from Lane et al. (1985) with a single step and 28 cycles of polymerase chain reaction (PCR), annealing at 53°C, using the HotStarTaq *Plus* Master Mix Kit (Qiagen, Valencia, CA, United States). Following the PCR, all the amplicon products from the different samples were mixed in equal concentrations and purified using Agencourt AMPure beads (Agencourt Bioscience Corporation, MA, United States). The purified PCR products were used to prepare a DNA library by following the Illumina TruSeq DNA library preparation protocol. All the samples were sequenced on the same MiSeq Illumina sequencer run (Illumina, San Diego, CA, United States) using MiSeq reagent kit V3 (Illumina) producing 2 × 300-bp long reads. PCR and sequencing were conducted in a commercial laboratory (MR DNA, Shallowater, TX, United States). All sequences were deposited in GenBank under SRA accession number PRJNA563840.

### Sequence Analysis

Sequence analysis were performed with the open-source software package DADA2 (v. 1.12) in “R” to model and correct Illumina-sequenced amplicon errors (Callahan et al., 2016). We applied the standard pipeline with the following parameters: trimLeft = 20, truncLen = c(290,250), maxN = 0, maxEE = c(2,2), truncQ = 2. Thus, the sequences were filtered, dereplicated, denoised by removing sample inference and chimeras, and merged. Representative sequences were classified against the SILVA v.128 database (Quast et al., 2012) for the taxonomy assignment and to obtain amplicon sequence variants (ASVs). To produce a finer taxonomical resolution, an additional BLAST search (Altschul et al., 1990) was performed on the ASVs that were the most abundant under each transplant condition. We obtained a total of 843,483 sequences for the entire datasets, which is an average of 17,000 sequences per sample.

### Statistical Analysis

Tests for normality of variance were performed using the Shapiro–Wilk test on R software (v3.4.3), which revealed that the data distribution was not normal for mortality and skeletal growth rates (*p* < 0.05). A multiple-comparison *non*-parametric Kruskal–Wallis test was used to analyze differences between the sites for each parameter investigated.

To compare the bacterial community composition and diversity, the sequence data were normalized by dividing counts by sample size to obtain a relative abundance. Possible differences in bacterial community composition were assessed by correspondence analysis (CA) with the vegan package in R (Oksanen et al., 2013). The Bray Curtis index (Bray and Curtis, 1957) and the Shannon diversity index (Shannon, 1948) were computed for all samples with the vegan package in R. To identify the ASVs that characterized the different samples, a simper test was applied with the plyr package in R.

## Results

### Coral Mortality

**FIGURE 1:**
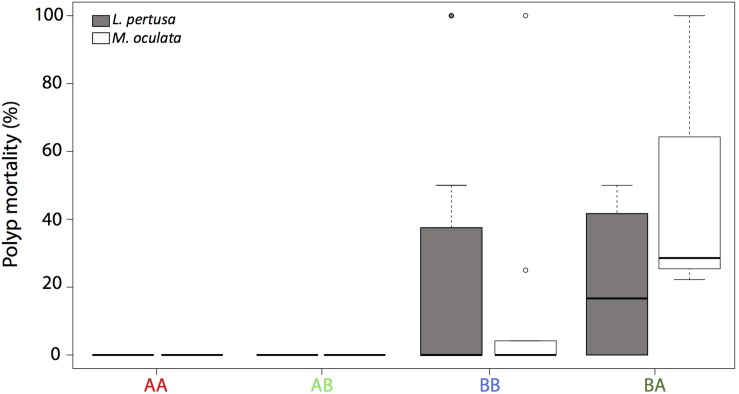
Polyp mortality of *L. pertusa* (gray) and *M. oculata* (white) at the deeper site A and the shallower site B. AA, coral originating from site A and redeployed at site A; BA, coral from site B and redeployed to A; AB, coral from site A and redeployed to B; BB, coral from site B and redeployed to B. The boxplots represent the median values, the quartiles, and the minimum and maximum.

For *Madrepora oculata*, polyp mortality was low when redeployed at their site of origin (Figure 1). For *L. pertusa* redeployed at their site of origin, there was no polyp mortality at site A, but some mortality at site B. There was no significant difference in mortality between species (*n* = 66, *p* > 0.05).

For *Lophelia pertusa*, there was no difference in mortality (average < 10%) between individuals that were cross transplanted from one site to another and those that were not (*n* = 23, *p* > 0.05). For *M. oculata*, there was a difference in mortality between the cross transplanted and *non*-cross transplanted corals (*n* = 43, *p* < 0.001) with a higher mortality (average 50%) for the corals that were moved from site B to A (Figure 1).

### Coral Growth Rates

**FIGURE 2:**
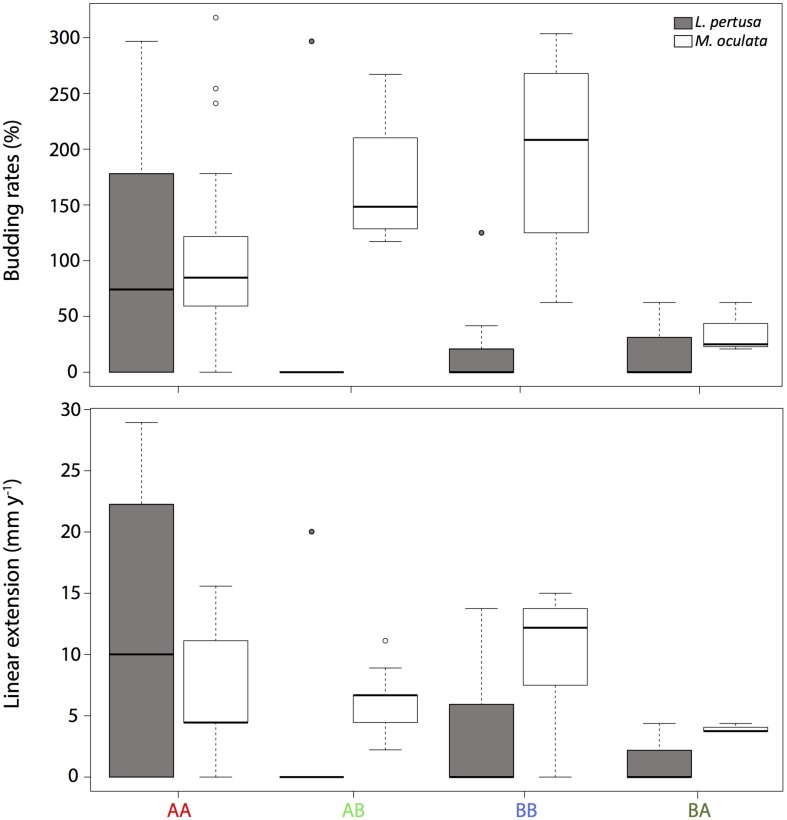
A Budding rates and B linear extension of *L. pertusa* (gray) and *M. oculata* (white) at the deeper site A and the shallower site B. AA, coral originating from site A and redeployed at site A; BA, coral from site B and redeployed to A; AB, coral from site A and redeployed to B; BB, coral from site B and redeployed to B. The boxplots represent the median values, the quartiles, and the minimum and maximum.

For each coral fragment, the budding rates and the linear extension were measured (Figure 2). Overall, *M. oculata* had a significantly higher budding rate than *L. pertusa* (*p* < 0.001).

For *Lophelia pertusa*, there was no significant difference between corals growing at the two sites, although the highest budding rate values (average of 104 ± 124%) were observed for corals that originated and remained at the deeper site A (Figure 2). For *M. oculata*, the corals that grew on the shallower site B (averages of 200 ± 80% and 170 ± 50% for BB and AB, respectively) had significantly higher budding rates than corals at site A (averages of 100 ± 90% and 36 ± 23% for AA and BA, respectively, *p* < 0,001) (Figure 2).

For the linear extensions, there was overall no significant differences between species. For *L. pertusa*, there was no significant difference between sites but the highest values (12 ± 13 mm y^–1^) were found at AA. For *M. oculata*, the *non*-cross transplanted corals at site B (mean of 10 ± 5 mm y^–1^ at BB) had significantly higher linear extension compared to corals from site A (mean of 6 ± 5 mm y^–1^ at AA, *p* < 0.05). However, there were no differences between cross transplanted and *non*-cross transplanted corals (Figure 2).

### Coral Bacterial Communities

**FIGURE 3:**
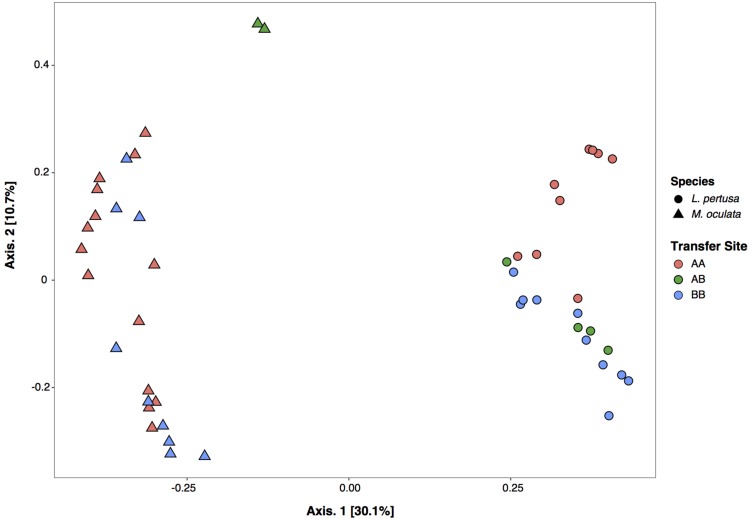
Correspondence analysis (CA) of bacterial community composition based on 16S rRNA gene for *L. pertusa* and *M. oculata*. AA, coral originating from site A and redeployed at site A; AB, coral from site A and redeployed to B; BB, coral from site B and redeployed to B.

The *in situ* experiment showed that *L. pertusa* and *M. oculata* had different bacterial community composition (ANOSIM *p* < 0.001) (Figure 3). *M. oculata*’s bacterial communities did not differ between the two sites A and B (ANOSIM *p* > 0.05). The *M. oculata* transferred from site A to site B seemed to have a different microbiome composition, but as only two samples were available, it could not be conclusive (Figure 3). Inversely, the bacterial communities associated to *L. pertusa* differed between the sites A and B (ANOSIM *p* < 0.05). The coral microbiome composition of *L. pertusa* transferred from site A to site B (AB) was similar to the bacterial community composition of the corals growing at site B (ANOSIM *p* > 0.05) (Figure 3).

**FIGURE 4:**
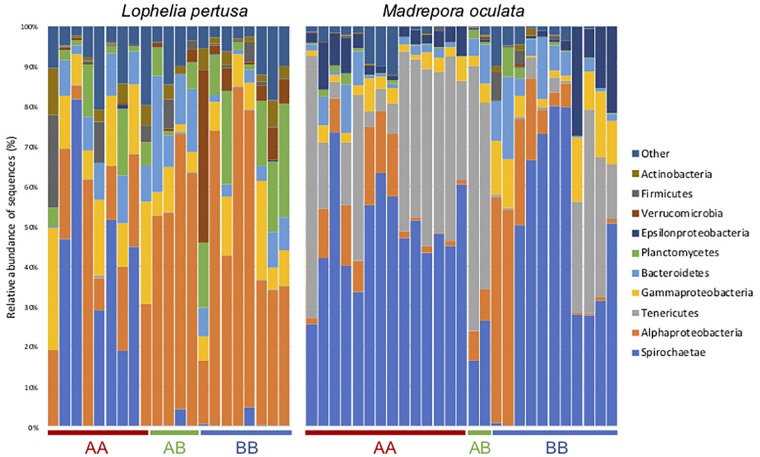
Bacterial community composition at the phylum/class level for *L. pertusa* and *M. oculata* from different transfer sites. AA, coral originating from site A and redeployed at site A; BA, coral from site B and redeployed to A; AB, coral from site A and redeployed to B; BB, coral from site B and redeployed to B. The 10 most abundant phyla are shown and the others are grouped as “Other.”

At the phylum level, the *M. oculata* bacterial communities were mostly dominated by *Spirochetae* (ASV1) and *Tenericutes* (ASV2) with no significant differences observed between sites (ANOVA, *p* > 0.05) (Figure 4). Inversely, there was a difference between sites for *L. pertusa*. The deeper site A coral microbiome was characterized by the phylum Spirochetae (ASV4) and the shallower site B by *Alphaproteobacteria* (ASV3). Interestingly, the bacterial communities of *L. pertusa* transferred from site A to B (AB) were composed of the phylum characterizing site B (Figure 4).

**FIGURE 5:**
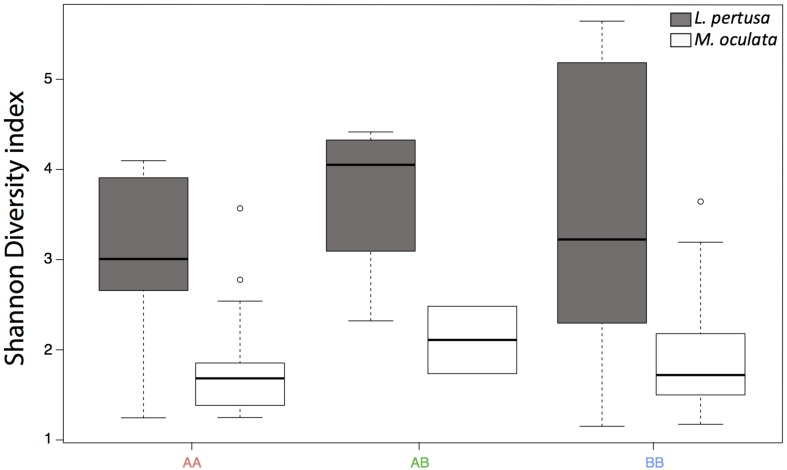
Shannon diversity index for *L. pertusa* (gray) and *M. oculata* (white) at the deeper A site and the shallower B site. AA, from site A and redeployed at site A; AB, from A and redeployed at B; BB, from B and redeployed at site B. The boxplots represent the median values, the quartiles, and the minimum and maximum.

The Shannon diversity index (Shannon, 1948) showed no statistical differences between the deeper and the shallower sites, but it was significantly higher in *L. pertusa* compared to *M. oculata* (*p* < 0.001) (Figure 5).

**FIGURE 6:**
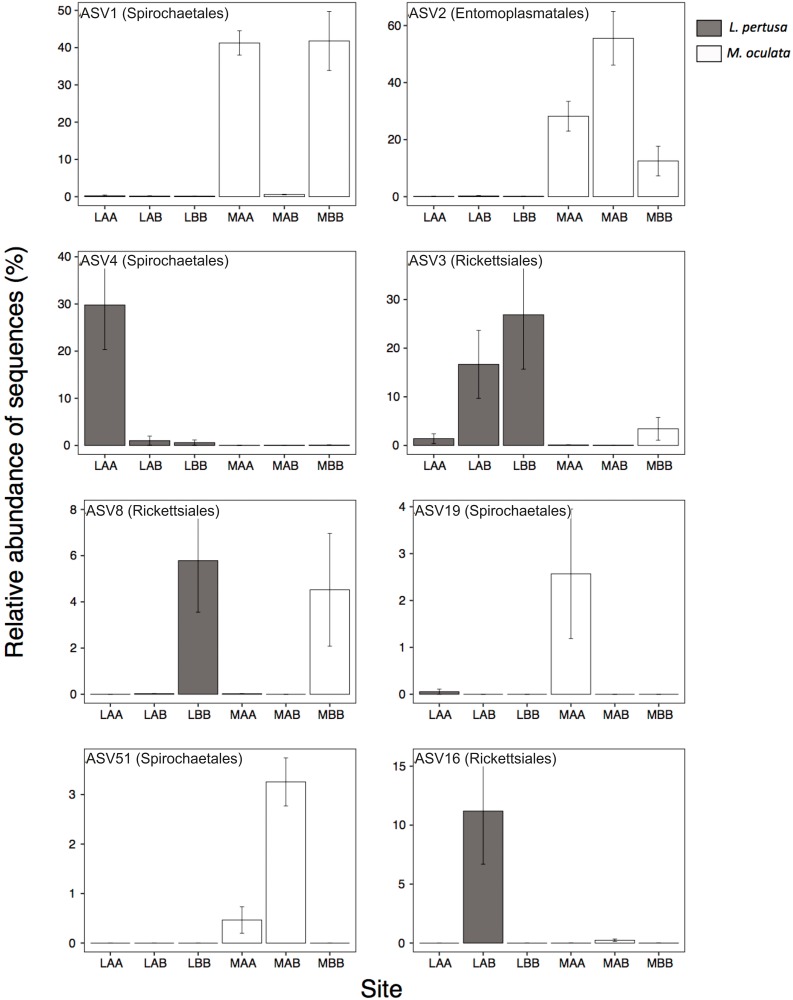
Relative proportion of the most abundant ASVs present in *L. pertusa* (gray) and *M. oculata* (white) for different transplant experiments (AA, AB, and BB). The affiliation of the ASVs at the order level is indicated in parentheses. A detailed taxonomic affiliation of each ASV is given in Supplementary Table S1. The mean ± SD is shown for each ASV.

We further identified at the ASV level the main bacteria present in each species and at each site by SIMPER analysis (Figure 6). The four most abundant ASVs represented more than 75% of the sequences in *L. pertusa* and *M. oculata*. The ASV1 (Order Spirochetales) and ASV2 (Order Entomoplasmatales) were significantly more abundant in *M. oculata* while ASV3 (Order Rickettsiales) and ASV4 (Order Spirochetales) were more abundant in *L. pertusa* microbiomes (Figure 6). Looking at sites, ASV4 appeared relatively more abundant at site A while ASV3 was dominant in corals growing at site B. Some of these ASVs were highly similar to sequences previously retrieved from CWCs and others were distantly related to sequences found in tropical corals (Supplementary Table S1). The ASV8 (Order Rickettsiales) was typical of the site B in both coral species (88% similarity to database sequences). The ASV19 (Order Spirochetales) was characteristic of *M. oculata* living at site A. ASV51 (Order Spirochetales) and ASV16 (Order Rickettsiales) were mostly present in the transplanted corals (AB) in both species. ASV16 was 100% similar to a sequence previously detected in *L. pertusa* (Supplementary Table S1). Finally, *Endozoicomonas*, a typical coral symbiont, was observed only in *L. pertusa* and *M. oculata* redeployed at their original site (Supplementary Figure S1).

## Discussion

Our results cast a new light on the strength of the host-microbiome relationship in CWCs. *Madrepora oculata*, conserved overall its bacterial community composition at the ASV level across habitats, although some bacteria were only present at one of the sites. Inversely, *L. pertusa* had a more variable bacterial community composition that changed with habitat. Interestingly, cross transplanted *L. pertusa* microbiomes became similar in composition to the bacterial communities of the local *L. pertusa* corals. Differences in bacterial community composition have been observed earlier for *L. pertusa* and *M. oculata* at large geographical scale (Jensen et al., 2019) but also between *L. pertusa* from the Mediterranean Sea and a Norwegian fjord (Yakimov et al., 2006; Neulinger et al., 2008). At a smaller geographical scale, there were differences in bacterial community composition within the Trondheimsfjord, but they were not related to the distance between sites (Neulinger et al., 2008). The present observations of the acquisition of a local microbial signature by *L. pertusa* is new, and it opens new hypothesis about the corals’ acquisition of a microbiome. The geographical differences could result from a horizontal transfer of the bacterial community. Such transfer could give a plasticity to some coral microbiomes, which could change according to environmental conditions. This has been shown with the comparison of *Eguchipsammia fistula* microbiomes between *in situ* and aquarium samples (Röthig et al., 2017). Aquarium conditions were also recently shown to transform *L. pertusa* microbiome within a day (Galand et al., 2018). Inversely, *M. oculata*’s microbiome was maintained for at least 6 months in aquaria (Galand et al., 2018). Here we thus validate *in situ* the previous observations made in aquaria, showing that *L. pertusa*’s bacterial community can quickly change with a changing environment. The acquisition of new microbes by *L. pertusa* may also be due to differences in nutrition that may lead to biochemical and/or metabolic differences, which in turn influence the selection of different microbial communities (Dodds et al., 2007; Schöttner et al., 2012; Meistertzheim et al., 2016). The difference in bacterial community observed between *L. pertusa* and *M. oculata* sharing the same habitat agrees with former observations for these coral species (Hansson et al., 2009; Meistertzheim et al., 2016).

Among the bacterial taxa detected in the present study, the *Entomoplasmatales* (phylum Tenericutes), which are characterized by the lack of cell walls, were previously observed in association with gorgonian corals where they dominate the assemblage (Gray et al., 2011). These bacteria were also observed in Atlantic *L. pertusa* and are supposed to play a major role for the coral (Neulinger et al., 2008; Kellogg et al., 2009). For *M. oculata*, we detected *Spirochetes*, which have not been seen earlier in this coral species. This bacterial association has already been observed with *L. pertusa* (Kellogg et al., 2009; Meistertzheim et al., 2016), *E. fistula* (Röthig et al., 2017) and with gorgonian corals from the deep sea (Gray et al., 2011; Lawler et al., 2016; van de Water et al., 2016).

Here, we also report that *L. pertusa* acquired the coral-associated *Rickettsiales* when transferred to the shallower geographic location (B site). This bacterial order has already been observed in different gorgonian and coral species (Casas et al., 2004; Gray et al., 2011; Klinges et al., 2019). This microbe was also widespread in both healthy and diseased tropical corals and may induce white band disease (Gignoux-Wolfsohn and Vollmer, 2015). Moreover, this *Rickettsiales*-like prokaryote from the *Alphaproteobacteria* group, is a Gram-negative microbe and can cause disease in invertebrates (Antonio et al., 2000). Thus, we can make the hypothesis these *Rickettsiales* may be opportunistic bacteria, with potential pathogens, that affect the coral development. However, considering that we are at the taxonomic level of the Order, this hypothesis has to be carefully considered.

Our work based on an *in situ* transplant experiment revealed difference between species following habitats. Combining microbiome results with measures of coral mortality and skeletal growth, we can suggest that *M. oculata* has a habitat preference, the shallower B site. In contrast, the versatile microbiome and the stable skeletal growth suggested that *L. pertusa* may not have such a strict habitat preference. *M. oculata* showed higher growth rates (i.e., budding rate and linear extension) at site B compared to site A, and its transplantation to site A reduced its growth and increased mortality. This is in accordance with field observations showing that site A (deeper) is dominated by *L. pertusa* reefs whereas the site B (shallower) is dominated by *M. oculata* reefs (personal observations). *Lophelia pertusa* did not show significant difference in growth rate between sites, even though the highest values were always observed at site A.

The different habitat preferences may be associated to the different physiological characteristics of the two species coupled to the different hydrological features defining the two sites in the canyon. *Lophelia pertusa* and *M. oculata* exhibit distinct physiological functions, including skeletal growth rates (Lartaud et al., 2014), feeding behavior and preferences (Tsounis et al., 2010; Galand et al., 2020), reproduction cycles (Waller and Tyler, 2005), but also distinct associated microbial communities (Meistertzheim et al., 2016). Different patterns of distribution between *L. pertusa* and *M. oculata* have been reported in the Atlantic and Mediterranean regions where *M. oculata* is the dominant species in the mid slope while *L. pertusa* is more abundant in the down slope (Foubert et al., 2011; Fabri et al., 2017; Boolukos et al., 2019). Gori et al. (2013) also reported that *M. oculata* was more abundant at depths shallower than 500 m in the Mediterranean canyons Cap de Creus and Lacaze-Duthiers. These previous results together with our new data suggest that *M. oculata* has a strong preference for shallower habitats. This may be linked to different temperature range tolerance between species with a higher sensitivity of *M. oculata* to lower temperatures (Naumann et al., 2014).

Both *Lophelia pertusa* and *Madrepora oculata* feed mostly on zooplankton, but it has been demonstrated that they do not share the same metabolism (Gori et al., 2014). Analyses of lipid compositions, and their nitrogen isotopic signatures, suggest that the two species adopt different feeding strategies (Kiriakoulakis et al., 2005; Galand et al., 2020). *Lophelia pertusa* is a more opportunistic suspended feeder, so its capacity to catch enough prey to sustain growth may be higher compared to *M. oculata* (Mueller et al., 2014). This could explain the more widespread occurrence of *L. pertusa* reefs. In addition, it has been shown that CWC capture rates depend on current regimes (Purser et al., 2010). The hydrology of the Lacaze Duthiers canyon is driven by episodic dense water shelf cascades that overflow the slope and provide food to sessile organisms as the CWCs (Ulses et al., 2008; Canals et al., 2009). However, the different morpho-bathymetric sites of the canyon are not influenced by the same water currents, and thus, the availability of food transported from the surface varies following the slope (Sanchez-Vidal et al., 2008). The current regimes in the Lacaze Duthiers canyon also resuspend sediments that can cover the living polyps (Durrieu de Madron et al., 2005). *Lophelia pertusa* has a great tolerance to sediments and drill cuttings (Larsson et al., 2013), and may thus thrive in impacted sites. Inversely, *M. oculata* may be more sensitive to suspended materials and may better develop at the head of the canyon on vertical cliffs, where strong currents wash away sediments. This is in accordance with earlier reports suggesting that *M. oculata* settles preferentially on areas with high topography and strong but irregular water current (Fabri et al., 2017).

## Conclusion

We identified, through a transplant experiment, changes in *L. pertusa*’s coral microbiome composition between two sites and the acquisition of a local coral microbial signature after transplant. This transplantation did not affect *L. pertusa*’s skeletal growth rates but the transfer induced the acquisition of bacteria in the shallower site. Inversely, *M. oculata*’s microbiome did not change with transplantation, but its skeletal growth was reduced. Our results suggest a higher plasticity of *L. pertusa* microbiome, which could explain its success across sites contrary to *M. oculata* which seem to harbor a habitat preference. Thus, in the context of ongoing climate change in Mediterranean Sea, both species may show different responses to changing environmental conditions.

## Data Availability Statement

The datasets generated for this study can be found in the PRJNA563840.

## Author Contributions

LC, NL, FL, and PG designed the study. LC, FL, and PG analyzed the data. LC, PG, NL, EP, and FL wrote the manuscript.

## Conflict of Interest

The authors declare that the research was conducted in the absence of any commercial or financial relationships that could be construed as a potential conflict of interest.
