# Supplementary material for: Local Variability in Microbiome Composition and Growth Suggests Habitat Preferences for Two Reef-Building Cold-Water Coral Species
Source: Front Microbiol. 2020 Feb 21;11:275. doi: 10.3389/fmicb.2020.00275 (PMC7047212; doi:10.3389/fmicb.2020.00275)
Supplement: Supplementary file 2 [file Data_Sheet_1.DOCX]

Supplementary figure 1: Number of sequences normalized of the ASVs *Endozoicomonas* present in *L. pertusa* (grey) and *M. oculata* (white) from different transfer sites (AA, AB and BB). The mean ± SD is shown for each ASV relative abundance.
